# Implementation of Influenza-like illness Sentinel Surveillance in Togo

**DOI:** 10.1186/1471-2458-14-981

**Published:** 2014-09-20

**Authors:** Issaka Maman, Kossi Badziklou, Essoya D Landoh, Afiwa W Halatoko, Talla N Nzussouo, Gabriel N Defang, Tsidi A Tamekloe, Pamela J Kennedy, Williams Thelma, Komlan Kossi, Zoulkarneiri Issa, Abiba B Kere

**Affiliations:** National Influenza Reference Laboratory, Institut National d’Hygiène, 26 QAD Rue Nangbeto, 01 Lomé-Sud, POBOX: 1396, Lome, Togo; Division de l’Epidémiologie, Ministry of Health, Lomé, Togo; Influenza Division, U.S. Centers for Disease Control and Prevention, Atlanta, GA USA; Virology Department, U.S. Naval Medical Research Unit No 3, Cairo, Egypt; McKing Consulting, Influenza Division, U.S. Centers for Disease Control and Prevention, Atlanta, GA USA

**Keywords:** Influenza, Human, Sentinel surveillance, Influenza-like illness (ILI), Lomé commune

## Abstract

**Background:**

The emergence of avian influenza A/H5N1 in 2003 as well as the pandemic influenza A (H1N1) pdm09 highlighted the need to establish influenza sentinel surveillance in Togo. The Ministry of Health decided to introduce Influenza to the list of diseases with epidemic potential. By April 2010, Togo was actively involved in influenza surveillance. This study aims to describe the implementation of ILI surveillance and results obtained from April 2010 to December 2012.

**Methods:**

Two sites were selected based on their accessibility and affordability to patients, their adequate specimen storage capacity and transportation system. Patients with ILI presenting at sentinel sites were enrolled by trained medical staff based on the World Health Organization (WHO) case definitions. Oropharyngeal and nasopharyngeal samples were collected and they were tested at the National Influenza Reference Laboratory using a U.S. Centers for Disease Control and Prevention (CDC) validated real time RT-PCR protocol. Laboratory results and epidemiological data were reported weekly and shared with all sentinel sites, Ministry of Health, Division of Epidemiology, WHO and CDC/NAMRU-3.

**Results:**

From April 2010 to December 2012, a total of 955 samples were collected with 52% of the study population aged between 0 and 4 years. Of the 955 samples, 236 (24.7%) tested positive for influenza viruses; with 136 (14.2%) positive for influenza A and 100 (10.5%) positive for influenza B. The highest influenza positive percentage (30%) was observed in 5–14 years old and patients aged 0–4 and >60 years had the lowest percentage (20%). Clinical symptoms such as cough and rhinorrhea were associated more with ILI patients who were positive for influenza type A than influenza type B. Influenza viruses circulated throughout the year with the positivity rate peaking around the months of January, May and again in October; corresponding respectively to the dry-dusty harmattan season and the long and then the short raining season. The pandemic A (H1N1) pdm09 was the predominantly circulating strain in 2010 while influenza B was the predominantly circulating strain in 2011. The seasonal A/H3N2 was observed throughout 2012 year.

**Conclusions:**

This study provides information on influenza epidemiology in the capital city of Togo.

## Background

Influenza-like-illnesses (ILI) is a significant source of morbidity and mortality worldwide [1]. The World Health Organization (WHO) estimates that globally influenza accounts for between 3 and 5 million severe cases and 250.000 to 500.000 deaths annually, with most deaths occurring among elderly populations [2].

In temperate regions, ILI is reported throughout the year with a marked increase in cases recorded during winter periods [3]. However, in tropical and subtropical regions where viral transmission occurs throughout the year, the data on the burden of influenza-like-illness are limited. Nevertheless there is some evidence of a slight increase in cases during the rainy season [4, 5].

The emergence of new highly pathogenic influenza A/H5N1 viruses in 2003 [6], their wide circulation in wild and domestic birds and its association with human infections which involves high mortality, has raised global concern about the risk of another influenza pandemic. The emergence of novel human pandemic influenza A (H1N1) in April 2009 [7, 8] and its rapid worldwide spread has motivated the monitoring of influenza and has enhanced preparedness to counter a possible emerging pandemic. In the African Region, countries in collaboration with international partners (e.g. WHO, CDC, NAMRU-3, etc.) put efforts together to establish influenza surveillance capacities as part of the broader strategy for Integrated Disease Surveillance and Response (IDSR) [9, 10]. While most countries in Asia, North America and Europe have well-established influenza surveillance, few such systems have been established in sub-Saharan Africa [5, 11]. Influenza surveillance helps in understanding the epidemiology and impact of the disease; therefore providing information about seasonality and the groups at high risk of influenza infection. Furthermore, the identification and characterization of circulating viruses will help to provide influenza isolates for monitoring changes in viral antigens and the development of vaccines. Thus influenza surveillance provides data for pandemic influenza monitoring and planning as well as for decision-making [12–14].

In Togo, the first suspected cases of human avian influenza A/H5N1 were reported between 2007 and 2008 in the Maritime Region, a few kilometers from the capital city Lomé, which has a population of more than 2 millions. Between April and December 2009, cases of ILI were observed and were suspected to be pandemic influenza given the emergence of the novel human pandemic influenza A (H1N1) pdm09. With no ongoing influenza surveillance, our country was not yet ready to confirm and effectively monitor the severity of the disease. The lack of molecular laboratory technology to detect influenza viruses significantly reduced our ability to manage and control the pandemic. Therefore, the Ministry of Health (MoH) in collaboration with the Institut National d’Hygiène (INH) decided to add influenza to the list of diseases with epidemic potential to be monitored and reported through the IDSR Program. By April 2010, Togo was actively involved in ILI surveillance with the support of United State Government through the Centers for Disease Control (CDC) and the Naval Medical Research Unit-3 (NAMRU-3).

This study aims to describe the implementation of ILI surveillance and results obtained from April 2010 to December 2012.

## Methods

### Study protocol

**Figure 1:**
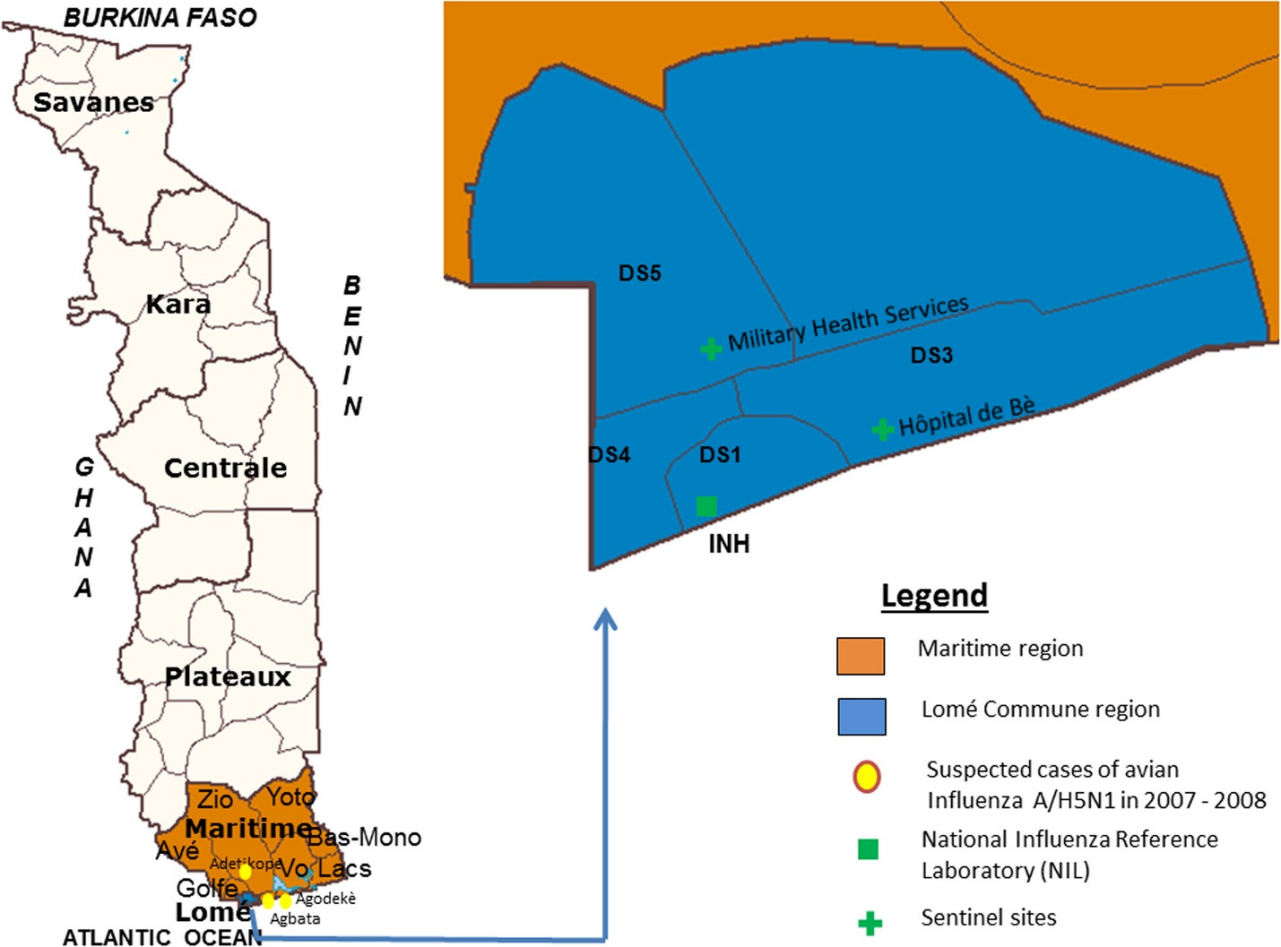
**Lomé commune region localization and institutions involved in the ILI sentinel Surveillance, Togo, April 2010-December 2012.** Lomé commune region is one of the six health region in Togo. Suspected cases of avian influenza A/H5N1 in poultry farms (yellow circles): In 2007 at Sigbéhoué (District des Lacs), Adétikope (District du Golfe) and Agodekê (District de Zio). In 2008 at Agbata (District des Lacs). All theses foci were located at few kilometers from the capital city, Lomé.

The ILI surveillance system constitutes a collaborative partnership between several Togolese institutions within the Ministry of Health (MoH). The departments involved in this surveillance are the Division of Epidemiology, the National Influenza Reference Laboratory (NIL) hosted by the Institut National d’Hygiène (INH), and the sentinel sites located at the Hôpital de Bè and Military Health Services in the capital city Lomé (Figure 1). A protocol for influenza surveillance was written with the technical support of CDC and NAMRU-3 experts.

### Sentinel sites selection

The ILI sentinel surveillance sites were selected based on their accessibility and affordability to patients with low socioeconomic status, the qualifications of medical staff, adequate specimen storage capacity, and an established transportation system to the National Influenza Reference Laboratory (NIL).

The first site was Hôpital de Bè, established in April 2010 and located in District N°3. This site was chosen for its geographical location in an area of high population density and high consultation rate. This hospital hosts a pediatric unit and a general medicine ward. The second site established in December 2011, is under the management of the Military Health Services and located in District N°5; its selection was based on the essential role of the Armed forces in case of a pandemic and their ability to serve both military and civilian populations. This military site is composed of three units and is attended by military personnel, their families as well as civilians.

The two sentinel sites are located in the capital city of Togo where approximately 20% of the country’s population lives. Lomé has two rainy seasons and two dry seasons: the long rainy season (April to June) and the short rainy season (mid-September to October). The long dry season extends from December through March, while the short dry season lasts for two months (July to August). Lomé is a coastal city that borders the Atlantic Ocean to the south, Ghana to the west, Benin to the east (Figure 1) and is at the crossroad with considerable commercial exchange of goods and movements of population.

### Case definition and study population

The WHO case definition [15] that was used, defined ILI as “any person with a sudden onset of fever (≥38°C) and cough or sore throat accompanied or not by general symptoms such as myalgia, prostration, headache or malaise”. This definition was used during 2010–2011 period. In 2012, the definition was changed to “any person with a sudden onset of fever (≥38°C) or history of fever and cough or sore throat accompanied or not by general symptoms such as myalgia, prostration, headache or malaise”.

At both sentinel sites, from Monday to Friday physicians enrolled the first two outpatients who met the case definition and samples were collected during consultation.

The study population included every outpatient, between April 2010 to December 2012, presenting at any of the sentinel sites and meeting the ILI case definitions regardless of age or sex and who consented to participate in the surveillance. This population represents a wide cross-section of ethnic and socioeconomic groups.

### Sample collection

Samples collected were nasopharyngeal and oropharyngeal swabs and were placed in the same tube containing a viral transport medium (VTM). They were stored between 2 to 8°C at the bacteriology laboratory of the sentinel site prior to delivery to the NIL within 48 hours. Before samples were transported, laboratory personnel at the sentinel site conducted quality control checks of information on patients’ case report forms. The NIL provides the sentinel sites with logistical and material support such as swabs, viral transportation media, cryovials, cool boxes, and ice packs. A quota of 20 samples was targeted from each sites and transported twice a week (Tuesday and Thursday) to the NIL. Review meetings with all stakeholders were organized two or three times per year as part of a strategy to improve the surveillance system by identifying strengths and areas of concern during these meetings.

### Data collection and management

Socio-demographic (age, sex, date of birth, residential area, travel history) and clinical (date of onset, date of consultation, previous treatment, vaccination status, co-morbidities) data were collected from all patients using a case report form (CRF) during consultation. Epidemiological data were stored in a single database with laboratory data using a single identification number for each patient. Each week, NIL provided reports on the distribution of total samples collected, as well as on the number of confirmed influenza cases to the MoH, to the sentinel sites, WHO FluNet, CDC, and NAMRU-3.

### Laboratory analysis

Samples collected were analyzed at the National Reference Influenza laboratory at INH. From every sample, three aliquots were made, two of which were stored at -80°C for external quality control and further analysis (if not subtyped) at NAMRU-3 in Cairo, Egypt. The other one was kept between 2 to 4°C for RNA extraction followed by influenza virus detection by real time RT-PCR within 72 hours after sample reception.

### RNA extraction and real time RT-PCR

For the testing of influenza viruses, RNA extraction was performed from 140 μl of naso and/or oro-pharyngeal cells contained in the VTM by using a QIAmp Viral RNA Mini Kit (Qiagen) following the manufacturer’s protocol. For detection and typing, it was run on an ABI 7300 machine, the real time RT-PCR using the Ambion enzyme AgPath one-step (Ambion, Applied Biosystems) that amplifies Influenza A and B. The U.S. CDC provided the protocol used to detect influenza viruses [16]. In order to determine the quality of the sample, the presence of human ribo-nucleoprotein (RNP) was assessed for each specimen tested.

### Statistical analysis

Socio-demographic and clinical epidemiological data were entered into a database created using Epi-Info Software version 3.5. Data analysis was conducted using SPSS software version 16.0 (SPSS Inc., Chicago, IL). Student t-test was used for comparison of mean age and the Pearson Chi-square or Fisher exact test to compare laboratory results by age groups and clinical symptoms.

### Ethical considerations

The protocol was approved by the MoH as part of the monitoring of diseases with epidemic potential and therefore did not require ethical review. Verbal consent was obtained from all patients.

## Results

### Characteristics of population

A total of 955 patients were enrolled in this study. Seven hundred and twenty seven (76%) patients were enrolled from the Hôpital de Bè and 228 (24%) from the Military Health Service site (Table 1). There was no significant difference in the proportion of females compared to males enrolled in this study (49.9% vs. 50.1%; p = 0.37), and the gender distribution at the two sites was similar. Most of patients (65%) were under 15 years of age, while less than 6% were 45 years or older. Patients who presented at the Hôpital de Bè were significantly older (mean age = 17.1 years) compared to those who were seen at the Military Health Service (mean age = 10.6 years with 71% of patients aged less than 5 years) (p = 0.0001). Approximately 2% of the patients reported having received influenza vaccination within the last year.

**Table 1:** **Characteristics of ILI patients enrolled into the influenza sentinel surveillance system, Togo, April 2010-December 2012**

	Hôpital de Bè n (%)	Military health Services n (%)	Total n (%)
**Number of subjects enrolled**	**228**	**955**	**727**
**Sex**			
Female	369 (50,8)	108 (47,4)	477 (49,9)
Male	358 (49,2)	120 (52,6)	478 (50,1)
**Age**			
Mean ± Std (yrs)	10,6 ± 13,2	15,7 ± 16,5	17,1±17,05
Median (range in yrs)	3 [1mo-58]	6 [1mo-90]	8 [1mo-90]
0-4	330 (45,3)	162 (71,0)	492 (51,5)
5-14	114 (15,7)	19 (8,3)	133 (13,9)
15-29	122 (16,8)	25 (10,9)	147 (15,4)
30-44	114 (15,7)	19 (8,3)	133 (13,9)
45-59	37 (5,1)	3 (1,3)	40 (4,2)
≥60	10 (1,4)	0 (0,0)	10 (1,0)
**Vaccination history (Self reported)**	3 (1,3)	17 (1,8)	14 (1,9)

Std = Standard mo = month yrs = years.

### Laboratory results

Of the samples collected for ILI surveillance, 236 (24.7%) tested positive for influenza viruses. Of these, 136 (14.2%) tested positive for influenza A virus and 100 (10.5%) for influenza B virus (Table 2). The proportion of influenza positive cases ranged from 32% in 2010 to 22.5% in 2012. Type and subtype varied by year, with influenza A being predominant in 2010 and 2012, and influenza B predominant in 2011. Of the influenza A subtypes, pandemic A (H1N1) pdm09 was predominant in 2010 while seasonal A/H3N2 was predominant in 2012 (Table 2). The proportion of influenza positive cases varied between different age groups with a higher proportion of influenza A detected in the 15–29 year-old group (20%) than other age groups (p = 0.01; Table 3). Significantly, the pandemic influenza A (H1N1) pdm09 was more often detected in patients aged 5–14 (p = 0.003) and 15–29 (p = 0.03) years than in other age groups. Seasonal A/H3N2 was predominant in patients aged 30–44 years (15%; p = 0.0003) and was the only influenza A subtype detected among patients who were 60 years or older.

**Table 2:** **Distribution of number of samples collected and influenza positivity rate by year, Togo, April 2010-December 2012**

					Subtypes of influenza A		
Years	Total n	Positive cases n (%)	Influenza B n (%)	Influenza A n (%)	A/H3N2 n (%)	A(H1N1) pdm09 n (%)	A/Unsubtypeable n (%)
**April to December 2010**	87	28 (32.2)	3 (3.4)	25 (28.7)	3 (3.4)	21 (24.1)	1 (1.1)
**2011**	335	88 (26.3)	49 (14.6)	39 (11.6)	17 (5.1)	20 (5.9)	2 (0.6)
**2012**	533	120 (22.5)	48 (9.0)	72 (13.5)	51 (9.5)	21 (3.9)	0 (0.0)
**Total**	**955**	**236 (24.7)**	**100 (10.5)**	**136 (14.2)**	**71(7.4)**	**62 (6.5)**	**3 (0.3)**

**Table 3:** **Distribution of influenza viruses confirmed and ILI patients by age, Togo, April 2010-December 2012**

						Subtypes of influenza A		
Age groups (Years)	Total n	Negatives n (%)	Positive cases n (%)	Influenza B n (%)	Influenza A n (%)	A/H3N2 n (%)	A(H1N1) pdm09 n (%)	A/Unsubtypeable n (%)
**0-4**	492	392 (79.7)	100 (20.3)	48 (9.7)	52 (10.6)	29 (5.9)	21 (4.3)	2 (0.4)
**5-14**	133	92 (69.2)	41 (30.8)	20 (15.0)	21 (15.8)	5 (3.7)	16 (12.0)	0 (0.0)
**15-29**	147	107 (72.8)	40 (27.2)	10 (6.8)	30 (20.4)	11 (7.5)	19 (12.9)	0 (0.0)
**30-44**	133	92 (69.2)	41(30.8)	17 (12.8)	24 (18.0)	20 (15.0)	3 (2.2)	1 (0.7)
**45-59**	40	28 (70.0)	12 (30.0)	5 (12.5)	7 (17.5)	4 (10.0)	3 (7.5)	0 (0.0)
**≥60**	10	8 (80.0)	2 (20.0)	0 (0.0)	2 (20.0)	2 (20.0)	0 (0.0)	0 (0.0)
**Total**	**955**	**719 (75.3)**	**236 (24.7)**	**100 (10.5)**	**136 (14.2)**	**71 (7.4)**	**62 (6.5)**	**03 (0.3)**

### Temporal distribution

**Figure 2:**
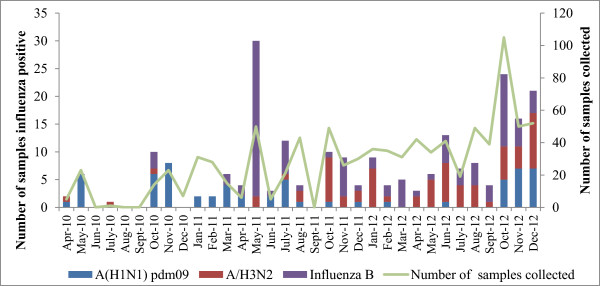
**Monthly distribution of number of samples collected and influenza viruses confirmed, Togo, April 2010-December 2012.** The influenza positivity rate was 26% in May and 30% in October and corralated with the presence of the pandemic influenza A (H1N1) pdm09 in 2010. In 2011 year, the influenza B became predominant and representing 93% of all viruses detected in May. During the same year, a second peak of ILI was observed and corralated with the seasonal influenza A/H3N2 in October. From this month, there was a co-circulation of influenza type A and type B.

ILI was observed throughout every year with irregular peak activity occurring twice annually during the months of; May and November in 2010; May and October in 2011; April/August, October in 2012 (Figure 2). However, the number of patients/samples enrolled was not consistent. Influenza A virus was detected predominantly in 2010 and correlated with the ILI peak. The first cases of pandemic influenza A (H1N1) pdm09 were only confirmed in May. During the ILI peak, the influenza positivity rate was 26% in May and 30% in November with the pandemic influenza strain, the most subtype detected. The pandemic virus remained predominant between October 2010 and April 2011 (Figure 2). During the ILI peaks in 2011, the influenza positive rate ranged from 20% to 60% with the predominance of influenza B virus activity in May representing 93% of all virus detected (28/30). The second peak was correlated to the seasonal influenza A/H3N2 activity in October with 80% of viruses detected (8/10). From October 2011, there was a co-circulation of influenza type A and type B with low activity of pandemic strain until September 2012 while the seasonal influenza A/H3N2 was detected throughout the year 2012.

### Clinical manifestations

Fever (85%), cough (87%), and rhinorrhea (76%) were the major symptoms for all age groups although sore throat (38%) and headaches (14%) were also recorded (Table 4). ILI patients who tested positive for influenza were more likely to present with cough (p = 0.004) and headaches (p = 0.03) but were less likely to present with difficulty breathing (p = 0.02) compared to those who tested negative for the influenza virus. Among symptoms, only cough was more common in patients testing positive for influenza A than those who tested positive for influenza B (p = 0.003). Rhinorrhea was more common in patients with seasonal A/H3N2 than in those with pandemic influenza A (H1N1) pdm09 (p = 0.0004).

**Table 4:** **Clinical symptoms among patients with influenza-like illness, by influenza virus laboratory test findings, Togo, April 2010-December 2012**

	Total n (%)	Negatives n (%)	Positive cases n (%)	Influenza B n (%)	Influenza A n (%)	A/H3N2 n (%)	A(H1N1) pdm09 n (%)	A/Unsubtypeable n (%)
**Clinical symptoms**								
**Fever**	813 (85.1)	614 (85.3)	199 (84.3)	88 (88.0)	111 (81.6)	59 (83.1)	49 (790.0)	3 (100)
**Cough**	834 (87.3)	615 (85.5)	219 (92.8)	87 (87.0)	132 (97.0)	68 (95.8)	61 (98.4)	3 (100)
**rhinorrhea**	725 (75.9)	541 (75.2)	184 (78.0)	80 (80.0)	104 (76.4)	63 (88.7)	39 (62.9)	2 (66.7)
**Sore throat**	367 (38.4)	265 (36.8)	102 (43.2)	47 (47.0)	55 (40.4)	24 (33.8)	28 (45.2)	3 (100)
**Headache**	141 (14.8)	96 (13.3)	45 (19.1)	15 (15.0)	30 (22.0)	18 (25.3)	12 (19.3)	0 (0.0)
**Difficulty of breath**	175 (18.3)	144 (20.8)	31 (13.1)	14 (14.0)	17 (12.5)	11 (15.5)	6 (9.8)	0 (0.0)
**Total**	**955**	**719 (75.3)**	**236 (24.7)**	**100 (10.5)**	**136 (14.2)**	**71 (7.4)**	**62 (6.5)**	**3 (0.3)**

## Discussion

Due to the lack of ILI surveillance in Togo, there was no information about the epidemiology of ILI or influenza viruses until 2010. The first samples collected were processed in May 2010 and the presence of pandemic influenza A(H1N1) pdm09 virus was confirmed in Togo one year after the novel pandemic influenza occurred in Mexico (April 2009). This is the first report that describes the epidemiology of influenza in Togo using data from the ILI sentinel surveillance system. During the two and half year period of ILI sentinel surveillance, influenza viruses were detected in 236 (25%) of 955 samples. The average percentage positive in this study was higher than the positivity rate observed in 15 other African countries between 2006 and 2010 [17]. However, during the same timeframe, other countries in the temperate climate region: Madagascar (40%), Morocco (27%) and South Africa (40%) did show a high positivity rate as well as Peru (35%; 2006–2008) [18].

There are several reasons to explain the difference in the percentage positive observed between countries including the temporal distribution of these viruses, the sample collection method, the number of samples collected and the geographical distribution of sentinel sites. Most of our data includes post pandemic influenza A (H1N1) pdm09; this is a different picture compared to other African countries (2006–2010) and to the South American region. The sample collection method was different from one country to another. In our study, we used two swabs (one oro-pharyngeal and one naso-pharyngeal) for each enrolled patient and put both swabs in the same cryotube, thereby increasing the viral load and enhancing RT-PCR detection while in some other countries samples were collected either with naso-pharyngeal [19] or oro-pharyngeal swab only [18, 20]. In addition, the number of samples tested in most of the other countries was quite high compared to our sample numbers and they were collected from many sites ranging from only 3 to as many as 22. We only used two sentinel sites as our catchment area. The influenza positivity rate varied by year with the highest rate obtained in 2010. Since the number of samples collected during this year was very low (87 samples) than the two subsequent years, the rate could be influenced. Nevertheless, our percentage positive was similar to that of Ghana and Rwanda in Africa [17] and that of Taiwan [21], but at different periods of time.

Influenza A was predominant in 2010 with pandemic influenza A (H1N1) pdm 09 in our study; this observation was similar to that of other countries in West Africa [17]. However, in 2010, the situation was different in other sub-regions with predominance of influenza B in Central/South and North Africa [17, 22] and seasonal influenza A/H3N2 in East Africa [17]. This difference could be explained by the fact that circulation of pandemic influenza A (H1N1) pdm09 was delayed in West Africa and occurred one year after it was predominantly circulating in other African sub-regions [17, 23]. While two years is not sufficient time for an adequate description of the seasonality of influenza virus transmission, we did observe trends in the Lomé commune region. The influenza B virus showed a peak activity during the rainy seasons (May and October) and the pandemic influenza A (H1N1) pdm09 was more frequent during the long dry season while the seasonal A/H3N2 was detected across both seasons. Although the seasonality of influenza viruses in African countries is not yet clear, we observed that our trends were similar with the influenza peaks, which have often been associated with the rainy season activity in other tropical countries [24–26].

In our study, Influenza cases were highest (30%) in the 5–14 year age group but also high among other age groups, with lowest percent positive (20.3%) among 0–4 and > 60 years (20.0%). This distribution is consistent with the observation in the study conducted in 15 countries of Africa during 2006 to 2010 and in Peru [17, 18, 20] in which young children and adults were shown to have the highest influenza viral disease. Contrary to our study, a study from Venezuela [27] showed higher detection rates in 0–4 year olds. The percentage positive of influenza A was significantly higher in ILI patients in the 15–29 age groups. Therefore, we found that pandemic influenza A (H1N1) pdm09 was detected significantly among 5–29 years old. This finding is consistent with other studies in the African region [17, 22, 28] that have found that pandemic influenza A (H1N1) pdm09 is most commonly identified in school-age children and young adults. While pandemic influenza A (H1N1) pdm09 appeared more often in older children, seasonal influenza A/H3N2 appeared more likely to infect adults in the 30–44 year-age category. Our finding was similar to the observation from a study conducted in Peru [18], where the author found that the seasonal influenza A/H3N2 virus was detected with adults of 45 to 59 years. In conclusion, our results are consistent with studies from Africa and South American regions which observed that seasonal influenza A/H3N2 affected a wide range of age groups with predominantly 30 to 60 years old while the pandemic influenza A (H1N1) pdm09 and influenza B virus infections occurred more frequently among older children and young adults.

We observed that clinical symptoms were associated with influenza viruses. The influenza type A was more frequently detected than type B in patients presenting with cough and rhinorrhea. This result is consistent with the observation of a study from Venezuela [27]. In contrast with this study were pandemic influenza A (H1N1) pdm09 was associated with ILI patients with cough, our study showed that ILI patients with rhinorrhea were associated with seasonal A/H3N2.

Our study had some limitations. Our data were collected only from 2 sites in an urban area in the capital city of Togo and could not be generalized to the population. The percent influenza positivity and age distribution of positive cases were influenced by the low number of samples collected which may be attributable to the non availability of a physician to collect nasal and orpharyngeal swabs. The low number of samples may have also contributed to the high positivity rate. In addition, this low proportion may not be representative to better describe the distribution of influenza cases in the age groups. Physician time limitations were due to the time consumption and their workload (number of patients viewed in consultation at the outpatients’ department). Children were over-represented in this study thus introducing a bias, as the number of adults was not comparable to children under 5 years old. Some possible reasons to explain this bias include the fact that the Military Health Service has three units but only the family health care center was functional when added as a site in December 2011. Because this unit is a pediatric health center, the high number of children enrolled from this site can account for the observed figures. At the Hôpital de Bè site, we observed that many patients, mostly adults were not enrolled as ILI patients due to the lack of recorded fever (≥38°C), suggesting that we should be considering history of fever as one of the enrollment criteria for ILI. This study focused exclusively on outpatients thus limiting our ability to examine the severity of the influenza viruses in hospitalized cases. Since our influenza surveillance system had challenges in collecting samples of severe acute respiratory infection (SARI) and the lack of data on hospitalizations with patient follow-up we excluded discussions on SARI from this study. To improve our influenza surveillance system, it will be necessary to expand the system in other regions by including SARI surveillance for severe disease to give a complete picture of influenza burden and epidemiology in our country.

## Conclusions

These data provided information on the epidemiology of influenza in the Lomé commune region in the capital city of Togo. Some efforts are needed to allow better understanding of influenza burden and epidemiology by expanding sentinel sites in other regions and including SARI surveillance. Future studies will also be focused on identifying the etiologic agents for the 75% of ILI cases that were negative for influenza viruses. Retrospective analyses of these stored samples will be necessary to identify other respiratory viruses circulating, including respiratory syncytial virus (RSV), coronaviruses, Human Metapneumovirus (HMPV) and rhinoviruses.

## Abbreviations

ILI: Influenza-like illness
WHO: World Health Organization
ISDR: Integrated Disease Surveillance and Response
INH: Institut National d’Hygiène
NIL: National Influenza Laboratory
CDC: Centers for Disease Control and Prevention
NAMRU-3: Naval Medical Research Unit 3.
